# The use of stem cells in the treatment of mastitis in dairy cows

**DOI:** 10.1038/s41598-024-61051-0

**Published:** 2024-05-06

**Authors:** Joanna Pokorska, Sebastian Sawicki, Julia Gabryś, Dominika Kułaj, Edyta Agnieszka Bauer, Anna Lenart-Boroń, Klaudia Bulanda, Marta Kuchta-Gładysz, Anna Grzesiakowska, Jerzy Kemilew, Patryk Mikołaj Barton, Olga Lasek, Monika Bugno-Poniewierska

**Affiliations:** 1 Department of Animal Reproduction, Anatomy and Genomics, University of Agriculture in Krakow, al. Mickiewicza 24/28, 30-059 Krakow, Poland; 2 Department of Microbiology and Biomonitoring, University of Agriculture in Krakow, al. Mickiewicza 24/28, 30-059 Krakow, Poland; 3 “Kemilew Stem Cells for Animals” Company, Warsaw, Poland; 4 Department of Animal Nutrition, Biotechnology, and Fisheries, University of Agriculture in Krakow, al. Mickiewicza 24/28, 30-059 Krakow, Poland; 5 Kietrz Agricultural Combine LLC, ul. Zatorze 2, 48-130 Kietrz, Poland

**Keywords:** Stem cells, Diseases, Infectious diseases

## Abstract

Mastitis is a multifactorial inflammatory disease. The increase in antibiotic resistance of bacteria that cause mastitis means that cattle breeders would prefer to reduce the use of antibiotics. Recently, therapies using mesenchymal stem cells (MSCs) from various sources have gained significant interest in the development of regenerative medicine in humans and animals, due to their extraordinary range of properties and functions. The aim of this study was to analyze the effectiveness of an allogeneic stem cells derived from bone marrow (BMSC) and adipose tissue (ADSC) in treating mastitis in dairy cattle. The research material consisted of milk and blood samples collected from 39 Polish Holstein-Friesian cows, 36 of which were classified as having mastitis, based on cytological evaluation of their milk. The experimental group was divided into subgroups according to the method of MSC administration: intravenous, intramammary, and intravenous + intramammary, and according to the allogeneic stem cells administered: BMSC and ADSC. The research material was collected at several time intervals: before the administration of stem cells, after 24 and 72 h, and after 7 days. Blood samples were collected to assess hematological parameters and the level of pro-inflammatory cytokines, while the milk samples were used for microbiological assessment and to determine the somatic cells count (SCC). The administration of allogeneic MSCs resulted in a reduction in the total number of bacterial cells, *Staphylococcus aureus*, bacteria from the *Enterobacteriaceae* group, and a systematic decrease in SCC in milk. The therapeutic effect was achieved via intravenous + intramammary or intramammary administration.

## Introduction

Mastitis, which occurs in two forms: subclinical and clinical is one of the diseases that generates significant economic losses in dairy cattle breeding. These losses include reduced milk production and quality, premature drying of cows, medical costs, and animal deaths^1^. Mastitis is caused mainly by staphylococci, streptococci, and enterobacteria and may be clinical or subclinical. Antibiotic therapy is usually used in the treatment of mastitis in ruminants, a practice which may be associated with increased antibiotic resistance in bacteria and partial transmission of the antibiotics in milk^2^. Developing alternative methods of treating and preventing the development of mastitis is crucial in the fight against this disease. Modern therapies should, among other things, enable increased mobilization of the animal immune system to fight pathogens and support rapid mammary gland tissue regeneration, allowing cows to return to milk production^3^.

Recently, there has been significant interest in therapies using mesenchymal stem cells (MSCs) from various sources. Friedenstein et al.^4^ identified MSCs as cells that can form colonies resembling bone tissue and fibroblasts. Since then, numerous studies have shown these cells to be characterized by spindle-shaped morphology, adherence to plastic, lack of hematopoietic markers, low immunogenicity, and multipotency, i.e., the ability to differentiate into specialized cell types with specific functions.

The extraordinary range of properties and functions of MSCs allows them to be used in biomedical research, particularly in the development of regenerative medicine in humans and animals. The most frequently performed therapies using MSCs are related to joint and bone diseases^5,6^, diabetes^7^, and wound healing^8^. Moreover, attempts have been made to develop therapies using MSCs to treat mastitis in dairy cattle. The unique properties of MSCs to modulate innate and adaptive immune responses could be crucial in the therapy of mastitis. In the first phase of the immune response, after the pathogen invasion, MSCs promote the activation and recruitment of neutrophils into the inflammation site and inhibit their apoptosis. In addition, MSCs promote macrophage migration, influx and activate NK cells, thereby the immune response intensifies at the site of inflammation and prevents it from spreading. Then MSCs promote the T lymphocytes proliferation and the migration of Th17 cells^9^. The first such experiments concerned the use of fetal bovine MSCs (FBSC)^10^, and more recently, MSCs have also been isolated from adipose tissue^11^ and the umbilical cord^12,13^. This study describes the effectiveness of an allogeneic suspension of stem cells derived from bone marrow (BMSC) and adipose tissue (ADSC) in the treatment of subclinical udder inflammation in dairy cows.

## Results

### Cultivation and differentiation of ADSCs

**Figure 1:**
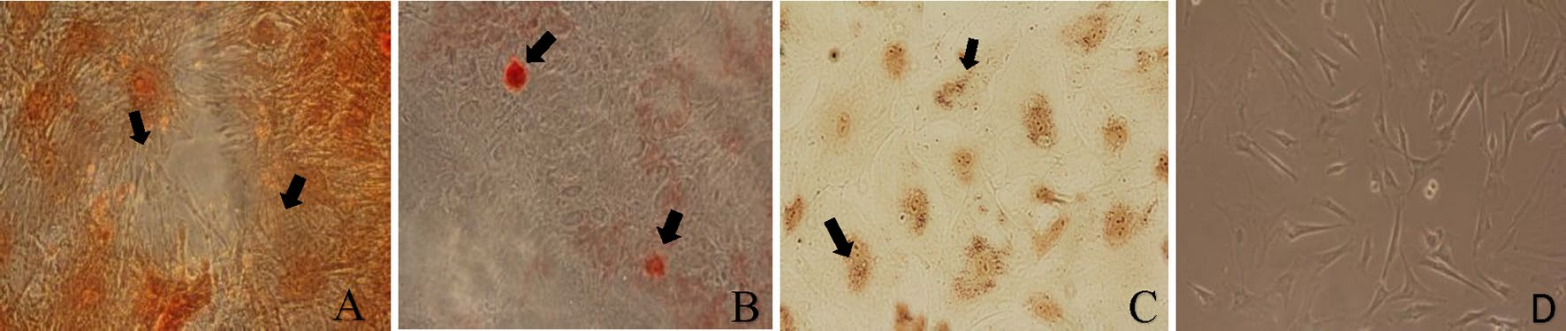
Results of chondrogenic, osteogenic, and adipogenic differentiation. (**A**) Safranin O staining after 21 days in chondrogenic differentiation medium with marked intracellular proteoglycans. (**B**) Alizarin Red staining of cells after 21 days in osteogenic differentiation medium with marked calcified extracellular matrix deposits. (**C**) Oil Red O staining after 14 days of culture in adipogenic differentiation medium with marked lipid droplets. (**D**) Fibroblast-like morphology of ADSCs.

Primary cell cultures were obtained following digestion with collagenase. Adhesion occurred approximately 48 h after seeding, and the isolated cells showed a fibroblast-like shape with a central nucleus and abundant cytoplasm. Subsequent passages were performed when 70–80% confluence was reached. Fibroblast-like cells were observed in all passages (Fig. 1).

After induction, trilinear differentiation was observed for ADSCs in specially prepared media. On day 14 of induction, fibroblast-like cells showed changes in morphology. ADSCs differentiated into adipocyte, osteocyte, and chondrocyte lineages. The use of dyes on the 21st day of induction enabled the identification of the structures characteristic of individual lines. The Oil Red O dye stained the lipid droplets, the Safranin O dye stained the intracellular proteoglycans, and the Alizarin Red dye stained the calcified deposits of the extracellular matrix (Fig. 1).

### Microbiological analysis of milk

The total bacterial count was used as a general assessment of the cow’s condition. The numbers of bacteria increased from day 0 (D0) and the highest values were observed on day 1 (D1) following the administration of stem cells, regardless of their origin. ADSCs seemed to be more effective in reducing the total bacterial count, as a significant decrease was observed at the subsequent time points (D3 and D7). The lowest average total bacterial count in milk was found following intravenous (ADSCs and BMSCs) and intravenous + intramammary (ADSCs) administration.

There were significantly fewer Gram-negative bacteria, i.e. *Enterobacteriaceae*, *E. coli* and ESBL-producing *E. coli*, compared to *S. aureus*, which prevailed throughout the experiment and seemed to be the primary cause of mastitis in this group of cows. The mean number of *E. coli* did not exceed 100 CFU/mL of milk.

*S. aureus* appeared in practically all samples of milk from cows with mastitis, with a mean range from 829.75 to more than 2700 CFU/mL on day 0 (D0). In the case of BMSCs, the highest numbers of *S. aureus* were reached after 24 h (D1), decreasing on day 3 (D3) and then increasing on day 7 (D7). When ADSCs were administered, the number of *S. aureus* systematically decreased until the third day (D3), and were then increased on day 7 (D7), but only to less than half of the initial (D0) value (Table 1).

**Table 1:** The influence of the stem cells on SCC and the number of bacteria in milk.

Parameter	Day	Type of suspension			General application p value
		BMSCs	ADSCs	NaCl	
Total bacterial count (CFU/mL)	D0	10,047.94 ± 22,170.51	75,663.16 ± 313,469.73	225.00 ± 386.22	0.024
	D1	20,013.13 ± 47,193.31	83,725.79 ± 343,052.65	192,925.00 ± 371,511	
	D3	4961.25 ± 9049.68	10,200.00 ± 18,609.79	3375.00 ± 5764.47	
	D7	60,578.13 ± 161,703.80	5300.56 ± 8140.38	3342.5 ± 4633.0	
*S. aureus* (CFU/mL)	D0	829.75 ± 1584.64	2751.74 ± 8468.11	62.00 ± 106.16	0.066
	D1	1224.38 ± 2928.26	890.89 ± 1769.97	150,350.00 ± 299,767.13	
	D3	458.50 ± 973.14	380.00 ± 736.12	285.00 ± 543.66	
	D7	850.31 ± 1120.31	1026.33 ± 2426.64	1062.50 ± 1405.60	
*MRSA* (CFU/mL)	D0	12.38 ± 27.66	13.37 ± 33.03	3.75 ± 6.85	0.004
	D1	5.44 ± 11.24	8.95 ± 19.79	23.75 ± 27.50	
	D3	4.13 ± 7.81	3.37 ± 10.93	32.50 ± 65.00	
	D7	4.00 ± 12.39^A^	21.39 ± 47.33^A^	1.75 ± 2.06	
*Enteriobacteriaceae* (CFU/mL)	D0	38.06 ± 102.26	81.63 ± 343.52	0	0.139
	D1	23.69 ± 56.96	82.95 ± 343.24	201.50 ± 399.01	
	D3	2.50 ± 5.62	79.26 ± 344.05	0	
	D7	22.13 ± 87.44	3.28 ± 6.07	1.25 ± 2.50	
*E. coli* (CFU/mL)	D0	25.63 ± 68.10	56.37 ± 228.63	0	0.612
	D1	20.94 ± 82.69	60.95 ± 251.69	176.25 ± 349.17	
	D3	2.69 ± 4.38	52.68 ± 229.40	0.75 ± 1.50	
	D7	19.00 ± 74.94	2.61 ± 5.37	1.00 ± 1.41	
SCC (1000/mL)	D0	736.25 ± 226.62^a^	571.58 ± 162.01^a^	239.25 ± 390.81	0.000
	D1	516.50 ± 212.84	393.74 ± 236.02	74.75 ± 32.11	
	D3	383.63 ± 237.07^b^	318.21 ± 216.29^b^	142.75 ± 177.66	
	D7	328.19 ± 166.34^c^	241.58 ± 142.88^c^	240.00 ± 126.69	

Values marked with the same letter differ significantly (A: p < 0.01; a–c: p < 0.05).

*ADSCs* allogenic adipose tissue stem cells, *BMSCs* allogenic bone marrow mesenchymal stem cells, *MRSA* methicillin-resistant *Staphylococcus* *aureus*, *SCC* somatic cell count.

As with the total bacterial count, intramammary and intravenous + intramammary administration seemed to be the most effective methods to reduce the number of pathogens (Table 2).

**Table 2:** The bacterial parameters and SCC in milk regarding an administration method and the stem cell type.

Parameter	Intravenous administration			Intravenous + intramammary administration			Intramammary administration			General application *p* value
	BMSCs	ADSCs	NaCl	BMSCs	ADSCs	NaCl	BMSCs	ADSCs	NaCl	
Total bacterial count (CFU/mL)	19,162 ± 15,762.82	110,803.51 ± 372,534.90	191,250 ± 372,534.90	13,409.04 ± 25,108.42	6142.92 ± 4671.19	567.50 ± 234.29	46,326.50 ± 51,688.27	3145.84 ± 2593.10	4025 ± 4871.77	0.024
*S. aureus* (CFU/mL)	1664.13 ± 1030.50	2976.19 ± 2917.56	16,025 ± 29,342.84	440 ± 195.20	514.90 ± 1146.83	127.50 ± 95.70	498.25 ± 295.15	172.67 ± 123.91	303.50 ± 342.42	0.066
*MRSA* (CFU/mL)	7.30 ± 5.27	13.55 ± 7.59	46 ± 52.33	3.58 ± 4.34	5.63 ± 5.84	14.75 ± 18.38	9.15 ± 10.22	15.54 ± 12.37	0.50 ± 0.61	0.000
*Enteriobacteriaceae* (CFU/mL)	0.35 ± 0.47	161.03 ± 106.80	201.25 ± 399.17	44.73 ± 101.74	5.33 ± 3.40	1.50 ± 3.00	1.70 ± 1.79	5.33 ± 3.40	–	0.139
*E. coli* (CFU/mL)	–	110.92 ± 73.70	176.50 ± 34	44.62 ± 26.84	2.16 ± 1.78	1.25 ± 2.50	1.05 ± 09	5.00 ± 4.22	0.13 ± 0.25	0.612
SCC (1000/mL)	556 ± 154.93	483.50 ± 133.48	86.50 ± 81.44	461.21 ± 167.96	327.75 ± 171.83	247 ± 211.01	462.20 ± 237.95	315.37 ± 146.44	93.50 ± 56.02	0.000

*ADSCs* allogenic adipose tissue stem cells, *BMSCs* allogenic bone marrow mesenchymal stem cells, *MRSA* methicillin-resistant *Staphylococcus aureus*, *SCC* somatic cell count.

The administration of BMSCs caused a progressive decrease in MRSA throughout the entire experiment, while the administration of ADSCs caused a decrease in MRSA until D3 and then a sudden increase on day 7 (D7). The number of MRSA at D7 was significantly lower in milk from cows that received BMSCs compared to those that received ADSCs (Table 1).

### Somatic cell count in milk (SCC)

The administration of stem cells (BMSCs, ADSCs) resulted in a successive reduction in the number of somatic cells in milk at subsequent time points (D1, D3 and D7). The therapeutic effect (reduction of the number of somatic cells to a level below 400,000/mL of milk in accordance with the Polish Standard PN-A-86002 and Regulation (EC) No. 853/2004 of the European Parliament and the Council) was achieved on the second day after administration of ADSCs (D1) and on the third day after administration of BMSCs (D3). The SCC in milk varied significantly depending on the type of stem cells administered (Table 1).

The best therapeutic effect related to reducing the number of somatic cells in milk was achieved with ADSCs using intravenous + intramammary or intramammary administration (Table 2).

### Analysis of blood hematological parameters and pro-inflammatory cytokines

There was an increase in the average number of leukocytes (WBC) and neutrophils (NEU) in the peripheral blood on the day after the administration of BMSCs, ADSCs, or NaCl (D1). At subsequent time points (D3, D7), there was a systematic decrease in WBC and NEU levels, to a level comparable to that on day 0 (D0). There was also a significant influence of stem cell type upon the level of WBC and lymphocytes (LYMPH) in the peripheral blood at D3 and D7 (Table 3).

**Table 3:** The influence of stem cell type on morphological parameters.

Parameter	Day	Type of suspension			General application *p* value
		BMSCs	ADSCs	NaCl	
WBC (G/L)	D0	8.48 ± 1.89	8.74 ± 2.63	8.06 ± 1.81	0.016
	D1	8.79 ± 2.71	9.66 ± 3.78	8.52 ± 1.80	
	D3	8.00 ± 1.77^a^	9.29 ± 3.49^a^	8.57 ± 1.28	
	D7	8.35 ± 2.09^b^	8.06 ± 1.93^b^	7.71 ± 1.07	
NEU (G/L)	D0	4.69 ± 1.84	4.93 ± 2.09	4.38 ± 0.98	0.054
	D1	4.92 ± 2.63	6.21 ± 3.54	8.42 ± 6.85	
	D3	4.07 ± 1.06	5.72 ± 2.97	4.39 ± 1.28	
	D7	4.85 ± 1.65	4.73 ± 1.46	3.99 ± 0.69	
LYMPH (G/L)	D0	2.51 ± 0.94	2.24 ± 0.89	2.39 ± 0.98	0.004
	D1	2.71 ± 1.72	2.16 ± 0.73	2.72 ± 1.06	
	D3	2.48 ± 1.03^c^	2.14 ± 0.75^c^	2.35 ± 0.55	
	D7	2.34 ± 1.06^d^	2.09 ± 0.95^d^	2.36 ± 0.26	
MONO (G/L)	D0	0.83 ± 0.53	1.07 ± 0.45	1.08 ± 0.56	0.000
	D1	0.75 ± 0.51	0.94 ± 0.46	1.12 ± 0.81	
	D3	0.93 ± 0.44	1.21 ± 0.37	1.51 ± 0.99	
	D7	0.81 ± 0.47	0.88 ± 0.50	1.10 ± 0.61	
IL-2 (ng/mL)	D0	0.303 ± 0.200	0.307 ± 0.272	0.474 ± 0.416	0.000
	D1	0.282 ± 0.193	0.425 ± 0.640	0.491 ± 0.345	
	D3	0.295 ± 0.282	0.313 ± 0.284	0.494 ± 0.334	
	D7	0.251 ± 0.231^e^	0.313 ± 0.292^e^	0.540 ± 0.429	
TNF- α (ng/mL)	D0	0.031 ± 0.023	0.026 ± 0.040	0.106 ± 0.098	0.000
	D1	0.038 ± 0.034	0.027 ± 0.042	0.091 ± 0.065	
	D3	0.031 ± 0.024	0.026 ± 0.060	0.084 ± 0.048	
	D7	0.047 ± 0.040 f.	0.023 ± 0.032 f.	0.106 ± 0.084	

Values marked with the same letter differ significantly (A: p < 0.01; a–f: p < 0.05).

*ADSCs* allogeneic adipose tissue stem cells, *BMSCs* allogeneic bone marrow mesenchymal stem cells, *WBC* white blood cells, *NEU* neutrophils, *LYMPH* lymphocytes, *MONO* monocytes, *IL-2* interleukin-2, *TNF-α* tumour necrosis factor α.

Intravenous administration was associated with statistically significant differences in the average number of WBCs (*p*—0.015) in the peripheral blood, depending on the type of stem cells used. There was also a significant difference in the number of monocytes (MONO, *p*—0.000) in peripheral blood after intravenous + intramammary and intramammary administration of stem cells (Table 4).

**Table 4:** The importance of blood parameters in relation to administration method and cell type.

Parameter	Intravenous administration			intravenous + intramammary administration			intramammary administration			General application *p* value
	BMSCs	ADSCs	NaCl	BMSCs	ADSCs	NaCl	BMSCs	ADSCs	NaCl	
WBC (G/L)	9.74 ± 0.61	9.71. ± 1.19	7.06 ± 0.19	8.83 ± 0.45	8.07 ± 0.64	7.46 ± 1.53	7.23 ± 0.54^a^	8.96 ± 0.49^a^	9.17 ± 0.71	0.016
NEU (G/L)	4.86 ± 0.45	6.24 ± 1.45	7.47 ± 7.42	4.77 ± 0.56	4.77 ± 0.39	3.82 ± 0.60	4.24 ± 0.56	5.10 ± 0.68	4.94 ± 0.62	0.054
LYMPH (G/L)	3.44 ± 0.63	2.54 ± 0.31	2.58 ± 0.86	2.37 ± 0.31	1.74 ± 0.14	1.81 ± 0.75	1.84 ± 0.13	2.15 ± 0.28	2.72 ± 0.39	0.004
MONO (G/L)	0.73 ± 0.16	0.65 ± 0.29	0.64 ± 0.29	0.77 ± 0.14^b^	1.18 ± 0.17^b^	1.78 ± 0.82	0.99 ± 0.13^A^	1.30 ± 0.08^A^	1.19 ± 0.26	0.000
IL-2 (ng/mL)	0.19 ± 0.07	0.25 ± 0.15	0.49 ± 0.11	0.21 ± 0.01	0.2 ± 0.01	0.22 ± 0.04	0.46 ± 0.02	0.58 ± 0.04	0.65 ± 0.04	0.000
TNF-α (ng/mL)	0.04 ± 0.01	0.01 ± 0.00	0.02 ± 0.01	0.03 ± 0.00	0.01 ± 0.00	0.05 ± 0.01	0.04 ± 0.01	0.05 ± 0.00	0.16 ± 0.03	0.000

Values marked with the same letter differ significantly (A: p < 0.01; a, b: p < 0.05).

*ADSCs* allogeneic adipose tissue stem cells, *BMSCs* allogeneic bone marrow mesenchymal stem cells, *WBC* white blood cells, *NEU* neutrophils, *LYMPH* lymphocytes, *MONO* monocytes, *IL-2* interleukin-2, *TNF-α* tumour necrosis factor α.

Although the administration of stem cells did not cause a sharp increase in pro-inflammatory cytokines (IL-6 and TNF-alpha) in the blood plasma, the values of these parameters differed significantly at D7, depending on the type of stem cells used (Table 3).

## Discussion

Mesenchymal stem cells have been used to treat many diseases (including the musculoskeletal system, liver, and kidneys) for some time^14^. The therapeutic benefit of MSCs results mainly from the effect of the extracellular paracrine factors they secrete upon neighboring cells and tissues, including promoting angiogenesis, modulating the immune response, supporting the renewal of the extracellular matrix, and stimulating progenitor cells to rebuild tissues that have been damaged due to disease or injury^15,16^. Additionally, MSCs create an anti-inflammatory environment, protecting against further development of infection and minimizing the risk of progression of cellular damage by secreting antimicrobial peptides (AMPs), such as cystatin C (CST3), elafin, hepcidin, cathelicidin-4 (CATHL4, indolicidin), lipocalin-2 (LCN2) or IDR-1, disrupting the integrity of bacterial cell membranes^17–21^. The anti-inflammatory effect of MSCs is also related to the inhibition of the proliferation of dendritic cells, natural killer cells, and T and B lymphocytes^22–26^.

The versatile properties of MSCs mean that they are increasingly used to treat diseases of companion and farm animals, especially dogs and horses^27^. The therapeutic use of MSCs has been shown to be safe, and this was confirmed here, as the hematological parameters and pro-inflammatory cytokine levels did not exceed physiological standards (Tables 3 and 4). The fluctuations in the levels of WBC and NEU on the first day after the administration of MSCs (BMSCs and ADSCs) were comparable to the changes in these indicators after the administration of saline, which most likely indicates that the animals experienced a local immune reaction to the injection. Ghai et al.^12^ and Peralta et al.^10^ indicated that local administration of allogeneic stem cells to animals did not induce a clinical response.

Recently, scientists have tried to use stem cells as an alternative to antibiotics for the treatment of mastitis^10,12,28^. The subclinical form of mastitis represents a larger problem, as it does not cause external symptoms, and its identification is based only on measuring changes in chemical parameters and the number of somatic cells in milk (SCC)^29,30^. The leading causes of mastitis are injuries and bacterial infections. Numerous studies on mastitis in dairy cattle have examined the predominant bacterial pathogens involved in the occurrence of this disease^31–34^. Its etiological agents can be classified into two categories, based on their origin: contagious (transmitted from cow to cow, such as *S. aureus* or *S. agalactiae*) or environmental pathogens (opportunistic pathogens such as *E. coli*, *S. uberis* or *Pseudomonas*, existing in the bedding)^35^. In the present study, the etiological agent of the cows appeared to be contagious, as *S. aureus* prevailed in the milk samples throughout the study. Reyher et al.^36^ showed that *S. aureus* is the most commonly isolated pathogen from the milk of cows with clinical mastitis and the second most commonly isolated pathogen from the milk of cows with subclinical forms of mastitis. While the treatment of *S. aureus* infections currently relies on antibiotic therapy, this method may become increasingly less effective, as resistance to β-lactam antibiotics is easily developed by this bacterium^37^. Moreover, antibiotic treatment does not promote the regeneration of mammary gland tissue, which appears essential for future milk production^28^. For this reason, searching for new strategies in fighting *S. aureus* infections seems to be more than justified. In our study, stem cell administration showed the reduction of culturable bacteria, including the most prevalent pathogen, *S. aureus*. Currently there are several studies on the antibacterial effect of stem cells in mastitis treatment. Cahuascanco et al.^28^ observed antiproliferative activity of bovine fetal mesenchymal stem cells on *S. aureus *in vitro and suggested that this antibacterial effect might be mediated by β-defensin and NK-lysine 1 activity. Peralta et al.^10^ also suggested that the administration of MSCs might be effective in the treatment of clinical mastitis caused by *S. aureus*.

While the number and type of bacteria in milk reflect the condition of the cow’s mammary gland, microbiological analysis requires laboratory infrastructure. A much simpler parameter for a cattle breeder to diagnose mastitis is the increased number of SCCs in milk^38^. During our experiment, we observed a systematic decrease in SCCs, which was most likely caused by the initiation of regenerative processes in the glandular tissue of the udder after the administration of stem cells. A similar reaction of the mammary gland to the administration of stem cells was demonstrated by Singh et al.^11^ and Ghai et al.^12^.

Our study has shown that both the origin of stem cells and their route of administration could influence the course of mastitis therapy. Administration of stem cells reduced SCC in milk. It was also shown that BMSCs had a more significant impact than ADSCs on reducing methicillin-resistant *S. aureus* (MRSA). In turn, ADSCs had a more significant impact on the reduction of *Enterobacteriaceae* and *E. coli*. Intravenous + intramammary and intramammary administration of stem cells resulted in the most significant decrease in the total number of bacteria in milk. Therefore, the choice of tissue from which stem cells are obtained for mastitis therapy, and the route of administration of these cells should take into account the types of pathogens detected in milk.

Despite the promising results, the use of stem cells in mastitis therapy requires further research, including studying a more significant number of animals and considering additional parameters such as physiological condition, animal age, and lactation phase, which may affect the course of mastitis and the distribution of stem cells in the body.

## Conclusion

This study has shown that even a single administration of MSCs had a beneficial effect, with a reduction in the number of mastitis-causing pathogens and decreased SCC in milk. To maintain the therapeutic effect, future studies should include multiple administrations of MSCs. Stem cell injections should be performed at a time appropriate to the animals' physiological condition and lactation phase, as they may affect the course of mastitis and the distribution of stem cells in the body. The therapies of bovine mastitis by mesenchymal stem cells are at the preliminary stage of research, so that makes it relatively expensive. The MSCs therapeutic potential allows us to conclude that in the future, milk producers may reduce economic losses related to antibiotics treatment, decreased milk production and cow culling.

## Materials and methods

### Experimental design

The experiment was carried out on 39 dairy cows of the Polish Holstein-Friesian breed, from a dairy production farm (Kietrz Agricultural Combine LLC—Pilszcz farm) in Poland. These animals were aged from 3.5 to 8 years, were in various stages of lactation, kept in a free-stall housing system, and fed a total mix ration. The average values of milk production characteristics before the start of the experiment were: milk yield, 37.61 ± 9.02 kg; fat, 4.05 ± 0.44%; protein, 3.52 ± 0.26%; lactose, 4.74 ± 0.21%; and SCC, 605.05 ± 258.18 (1000/mL).

The cows participating in the experiment were divided into two groups: experimental (n = 36; cows with a subclinical form of mastitis) and control (n = 3; healthy cows). They received a single dose of allogeneic stem cell suspension (experimental group) or physiological saline (control group). The experimental group was further divided into subgroups according to the method of administration of the solution: intravenous (n = 12), intramammary (n = 12), intravenous + intramammary (n = 12), and according to the type of allogeneic stem cells administered: bone marrow stem cells (BMSCs, n = 18) and adipose tissue-derived stem cells (ADSCs, n = 18). The therapeutic doses were applied just into the teat canal (intramammary) and into the jugular vein (intravenous). Blood and milk were collected from each cow at the following time intervals: before the administration of stem cells or saline (D0), and 24 h (D1), 72 h (D3), and 7 days (D7) after the administration of the solution. Blood was collected to determine hematological parameters and the level of pro-inflammatory cytokines, while milk was collected for microbiological assessment and to determine the number of somatic cells.

All experimental protocols and all methods were carried out in accordance with relevant guidelines and regulations, and were accepted by the 2nd Local Ethical Committee in Krakow (permit number 160/2021).

### Bone marrow stem cells—BMSCs

A commercial company operating in Poland (“Kemilew Stem Cells for Animals” Company, Warsaw, Poland) provided the bone marrow-derived stem cells. These were isolated from year-old donor heifers which had received pharmacological stimulation with recombinant granulocyte colony-stimulating factor (rh-G-CSF; filgrastim) to mobilize progenitor cells into the peripheral blood. The peripheral blood was collected into 450 mL sterile bags containing preservative fluid (CPDA-1), and then subjected to apheresis to isolate the white mononuclear blood cell fraction. Cell suspensions for injection were prepared in concentrations appropriate for the experimental system.

### Isolation, cultivation of MSCs from adipose tissue (ADSCs)

The ADSCs were isolated from adipose tissue from the peri-uterine region of adult cows of unregistered age and breed. The tissue was collected post-mortem at a slaughterhouse as part of commercial cattle slaughter following European Union regulations. Approximately 30 g of tissue was secured in a 50 mL Falcon tube containing sterile phosphate-buffered saline (PBS) supplemented with 2% penicillin/streptomycin solution (P/S; Sigma-Aldrich) and 0.1% amphotericin (Sigma-Aldrich), and transported to the laboratory at 4 °C, further analysis within 4 h of collection.

After washing several times with sterile PBS supplemented with 2% P/S and 0.1% amphotericin, then twice with Dulbecco’s Modified Eagle's Medium (DMEM, 4.5 g/L glucose, l-glutamine, and sodium bicarbonate, phenol red) (Sigma-Aldrich), the adipose tissue was digested in enzyme medium (1.5 mg/mL collagenase type I (ThermoFischer Scientific) in DMEM ((4.5 g/L glucose, l-glutamine, and sodium bicarbonate, phenol red; Sigma-Aldrich), supplemented with 1% P/S, and 0.1% amphotericin), which had been filtered through a 0.2 μm filter (Corning). The adipose tissue was digested for 3 h at 37 °C in an orbital shaker at 30 rpm. The cell suspension was then passed through a 70 μm filter (Corning) to remove undigested tissue debris and centrifuged at 400×*g* for 10 min. The supernatant was discarded, and the cell pellet was washed twice with sterile PBS before being resuspended in sterile culture medium (DMEM 4.5 g/L glucose, l-glutamine, and sodium bicarbonate, phenol red) (Sigma-Aldrich) supplemented with 10% fetal bovine serum (FBS, ThermoFisher Scientific), 1% P/S and 0.1% amphotericin. Then, the adipose cell suspension was distributed between four culture dishes covered with Geltrex™ (Gibco, ThermoFisher Scientific) and cultured at 37 °C in a humidified atmosphere containing 5% CO_2_.

After 48 h, non-adherent cells were removed, and fresh medium was added. The medium was changed every 2–3 days. Cells were then passaged (P1) at 70–80% confluence using trypsin–EDTA (0.25%) (ThermoFisher Scientific) and plated at a 1:3 ratio.

After reaching passage 3 (P3), cells were frozen at a final concentration of 1 million cells/mL in FBS (ThermoFisher Scientific) with 10% dimethyl sulfoxide (Sigma-Aldrich), by cooling to − 80 °C before transfer into liquid nitrogen for long-term storage.

Cryopreserved cells were thawed and expanded in DMEM supplemented with 10% FBS (ThermoFisher Scientific), 1% P/S, and 0.1% amphotericin. At 70% confluence, cells were allowed to differentiate into mesodermal lineages (chondrogenic, osteogenic, and adipogenic), and cells from this passage (P3) were used to prepare the therapeutic dose.

### Differentiation process of ADSCs and BMSCs

#### Adipogenic, osteogenic, and chondrogenic differentiation was performed in a monolayer

After thawing, cells were passaged (P3) in 24-well plates at a density of approx. 2 × 10^3^ cells/well and grown in DMEM supplemented with 10% FBS, 1% P/S, and 0.1% amphotericin and cultured at 37 °C in a humidified atmosphere containing 5% CO_2_. The medium was changed every 2–3 days. After reaching ~ 70% confluence, the cells were washed with sterile PBS and the medium was changed to either: adipogenic differentiation medium ((DMEM 1 g/L glucose, l-glutamine and sodium bicarbonate, without phenol red) (Sigma-Aldrich) supplemented with 2% FBS, 1% P/S, 10^−7^ M dexamethasone (Sigma-Aldrich), 1:100 (v/v) insulin-transferrin-selenium (ITS) (ThermoFisher Scientific), 0.5 mM 3-isobutyl-1-methylxanthine (Sigma-Aldrich) and 100 μM indomethacin (Sigma-Aldrich)); osteogenic differentiation medium ((DMEM 1 g/L glucose, l-glutamine and sodium bicarbonate, without phenol red) (Sigma-Aldrich) supplemented with 2% FBS, 1% (v/v) ITS, 1% P/S, 10^−7^ M dexamethasone, 50 μg/mL ascorbic acid (l-ascorbic acid-2-phosphate) (Sigma-Aldrich), 10 μL/mL 1M β-glycerophosphate (Sigma-Aldrich) and 0.1% amphotericin); or chondrogenic differentiation medium ((DMEM 4.5 g/L glucose, l-glutamine, sodium pyruvate, and sodium bicarbonate, with phenol red) (Sigma-Aldrich) supplemented with 2% FBS, 1% (v/v) ITS, 1% P/S, 10^−7^ M dexamethasone, 50 μg/mL ascorbic acid, 10 ng/mL TGF-β3 (recombinant human TGF-β3) (Sigma-Aldrich), and 0.1% amphotericin). The differentiation medium was changed every 3 days for 21 days. Confirmation of differentiation was performed using staining with Oil Red O (Sigma-Aldrich), 0.1% Safranin O (Sigma-Aldrich), and 2% Alizarin Red, pH 4.2 (Sigma-Aldrich).

The differentiation process of BMSCs was the same as the ADSCs.

#### Therapeutic dose

The allogeneic stem cells (BMSCs, ADSCs) were suspended in physiological saline, and therapeutic doses were prepared at a concentration of 50 million cells/mL for intravenous administration and at a concentration of 4 million cells/mL for intramammary administration. The prepared stem cell suspensions were stored at 4 °C for ± 12 h until use.

#### Microbiological milk analysis

The milk samples for the microbiological and cytological analysis were collected from the complete milking in accordance with the guidelines of the National Mastitis Council.

The bacteria from milk were identified and counted using the plate streak method. First, based on preliminary analyses we observed a large number of some groups of bacteria present in milk from cows with mastitis. Thus, in order to allow for a precise assessment of the numbers of colony forming units, a series of dilutions of 1 mL milk was prepared in a sterile 0.85% NaCl solution, starting from 10^−1^ up to 10^−5^. Both the undiluted fresh milk and its serial dilutions were streaked on Trypticase Soya Agar, MacConkey agar, Tryptone Bile X-Glucuronide agar, Baird Parker agar, Chromogenic ESBL agar, Chromogenic MRSA agar, to enumerate total bacteria, Gram-negative bacteria, *E. coli*, *Staphylococcus* spp., ESBL (extended spectrum beta lactamases) producing *Enterobacteriaceae* and methicillin resistant staphylococci, respectively. The plates were incubated aerobically at 37 °C for 48 h, and the colonies, characteristic for each media used, were counted. The colony counts obtained from all serial dilutions were used to calculate the colony-forming units per 1 mL of milk. ESBL was not considered in the statistical analyses because these bacteria were found in only one milk sample.

#### Analysis of blood hematological parameters and pro-inflammatory cytokines

The blood was collected into 10 mL tubes with EDTA (ethylenediaminetetraacetic acid) which 3 mL was used to assess the hematological parameters and 7 mL was centrifuged at 1000×*g* (5 min) to determine the cytokine levels (plasma). Hemograms were prepared by a commercial veterinary laboratory (VetLab, Poland). Cytokine determinations (TNFα and IL-2) were performed by enzyme-linked immunosorbent assay (ELISA) using ready-made reagent kits (Invitrogen), according to the manufacturer's recommendations. The absorbance of the samples was measured using an EPOCH 2 microplate spectrophotometer with Gen5 software (BioTek Instruments, INC, Highland Park, USA).

#### Somatic cell count in milk (SCC)

Two parallel smears of a milk sample were made on a degreased glass slide, which was then dried at room temperature, degreased in xylene, fixed in ethyl alcohol, and stained. The preparations were stained in the following reagents: May-Grünwald full, May-Grünwald 50% (v/v), and Giemsa 46% (v/v) (Merk). The slides were analyzed at 400× magnification in a Delta Optical light microscope. Cells were counted in 20 microscope fields. The number of somatic cells in 1 mL of milk was calculated as the quotient of the sum of the number of cells from all fields assessed and the f factor resulting from the microscope optics.

### Statistical analysis

Data were analysed using the Mann–Whitney *U* test, a non-parametric test of unpaired data from two samples. The computations for the Mann–Whitney *U* test gives the most accurate estimates of significance, especially with a small samples and/or when the data do not approximate a normal distribution^39^.

For comparing two small sets of observations *U* is determined:

$${U}_{1}={n}_{1}{n}_{2}+0.5{n}_{1}\left({n}_{1}+1\right)-{R}_{1}$$

$${U}_{2}={n}_{1}{n}_{2}+0.5{n}_{2}\left({n}_{2}+1\right)-{R}_{2}$$

$${U}_{1}+{U}_{2}+{n}_{1}+{n}_{2}$$

where *n*_*1*_—the number of observations from the first population, *n*_*2*_—the number of observations from the second population, *R*_*1*_—the sum of observation ranks for the first population, *R*_*2*_—the sum of observation ranks for the second population.

#### Test post hoc

The Least Significant Difference (LSD) calculates the smallest significant between two means as if a test had been run on those two means (rather than on all of the groups together). This enables direct comparison between two means from two individual groups. Any difference larger than the LSD is considered a significant result^40^.

The formula for the least significant difference is:

$${LSD}_{A,B}= {t}_{0.05/2,DFw}\sqrt{MSW\left(\frac{1}{{n}_{A}}+\frac{1}{{n}_{B}}\right)}$$

where: *t*—critical value from the t-distribution table with *α* = 0.05 and *DF*_*w*_ is the degrees of freedom within groups from the test table, *MSW*—mean square within, obtained from the test results, *n1, n2*—the sample sizes of each group.

## Data Availability

Data may be available upon request by contacting the corresponding author: P.J. email: joanna.pokorska@urk.edu.pl.
